# An Improved 2-Aminoimidazole Based Anti-Biofilm Coating for Orthopedic Implants: Activity, Stability, and *in vivo* Biocompatibility

**DOI:** 10.3389/fmicb.2021.658521

**Published:** 2021-04-21

**Authors:** Guglielmo Attilio Coppola, Jolien Onsea, T. Fintan Moriarty, Dirk Nehrbass, Caroline Constant, Stephan Zeiter, Merve Kübra Aktan, Annabel Braem, Erik V. Van der Eycken, Hans P. Steenackers, Willem-Jan Metsemakers

**Affiliations:** ^1^KU Leuven – Department of Chemistry, Laboratory for Organic & Microwave-Assisted Chemistry (LOMAC), Leuven, Belgium; ^2^KU Leuven – Department of Microbial and Molecular Systems, Centre of Microbial and Plant Genetics (CMPG), Leuven, Belgium; ^3^Department of Trauma Surgery, University Hospitals Leuven, Leuven, Belgium; ^4^KU Leuven – Department of Development and Regeneration, Leuven, Belgium; ^5^AO Research Institute, Davos, Switzerland; ^6^KU Leuven – Department of Materials Engineering (MTM), Biomaterials and Tissue Engineering Research Group, Leuven, Belgium; ^7^Peoples’ Friendship University of Russia, Moscow, Russia

**Keywords:** antibiofilm, titanium, implants, 2-aminoimidazole, coating, *Staphylococcus aureus*

## Abstract

Orthopedic device-related infections remain a serious challenge to treat. Central to these infections are bacterial biofilms that form on the orthopedic implant itself. These biofilms shield the bacteria from the host immune system and most common antibiotic drugs, which renders them essentially antibiotic-tolerant. There is an urgent clinical need for novel strategies to prevent these serious infections that do not involve conventional antibiotics. Recently, a novel antibiofilm coating for titanium surfaces was developed based on 5-(4-bromophenyl)-*N*-cyclopentyl-1-octyl-1*H*-imidazol-2-amine as an active biofilm inhibitor. In the current study we present an optimized coating protocol that allowed for a 5-fold higher load of this active compound, whilst shortening the manufacturing process. When applied to titanium disks, the newly optimized coating was resilient to the most common sterilization procedures and it induced a 1 log reduction in biofilm cells of a clinical *Staphylococcus aureus* isolate (JAR060131) *in vitro*, without affecting the planktonic phase. Moreover, the antibiofilm effect of the coating in combination with the antibiotic cefuroxime was higher than cefuroxime treatment alone. Furthermore, the coating was successfully applied to a human-scale fracture fixation device resulting in a loading that was comparable to the titanium disk model. Finally, an *in vivo* biocompatibility and healing study in a rabbit osteotomy model indicated that these coated implants did not negatively affect fracture healing or osteointegration. These findings put our technology one step closer to clinical trials, confirming its potential in fighting orthopedic infections without compromising healing.

## Introduction

Implanted devices are extensively used in orthopedic and trauma surgery to restore function and aid healing of broken bones. These interventions offer more rapid and accurate restoration of function and greatly improve the quality of life for the affected patient. Nonetheless, orthopedic device-related infection (ODRI) represents a major threat to the success of these surgical interventions. The incidence of ODRI ranges from 1 or 2% in case of elective joint replacement (Tande and Patel, 2014) up to 30% in complex open fractures where the protective barrier of the skin is breached (Papakostidis et al., 2011). *Staphylococcus aureus* and *Staphylococcus epidermidis* are the major pathogens in ODRI, accounting for more than half of all ODRIs (Moriarty et al., 2016).

The surface of the implant in fact serves as a substrate for bacterial attachment and the formation of biofilms, which are significantly more tolerant to antibiotics. The bacteria within the biofilm produce an extracellular polymeric substance (EPS), which can serve as a matrix that limits the penetration of antimicrobials and host immune cells into the biofilm. Therefore, intravenous antibiotic therapy alone is rarely successful in treating biofilm infections. Furthermore, exposure to low antibiotic concentrations exerts selective pressure which can lead to resistance development (Liu et al., 2011; Stanton et al., 2020).

ODRI imposes a significant burden on patients and healthcare systems due to the need for surgical revisions, long duration of antibiotic therapy, functional loss, and, sometimes, even the need for salvage procedures such as amputation of the affected limb or establishment of a continuous fistula (Metsemakers et al., 2016a). As a result, there is clear clinical need for innovative infection prevention strategies that do not rely on conventional antibiotic therapy. In this context, local delivery systems, such as antibacterial coatings or antibiotic loaded biomaterials have emerged as attractive means to support improved infection prevention (Metsemakers et al., 2020). Currently, most orthopedic implants are made out of metals such as stainless steel or titanium alloys, which lack an active antimicrobial surface that can prevent biofilm formation (Zhang et al., 2014; Metsemakers et al., 2016b). In addition to containing antibacterial components that are effective in preventing bacterial colonization and biofilm formation, active antimicrobial coatings should be biocompatible and should not elicit a significant foreign-body response. Furthermore, such a coating should not interfere with bone healing, and should display sufficient mechanical stability, so that it does not detach from the surface when placed under mechanical stress, as may be experienced during implant placement (Zhang et al., 2014). Finally, in times where antibiotic resistance is acknowledged as a worldwide problem, the development of non-antibiotic coatings seems crucial (Metsemakers et al., 2021). Numerous coatings have been developed over the years containing various bactericidal materials and molecules, such as silver-coated implants, implants coated with disinfectants like chlorhexidine and iodine, and antibiotic-coated implants (Zhang et al., 2014; Kuehl et al., 2016). Nonetheless, novel strategies focusing on biofilm formation and dispersal are gaining attention as their integration with conventional antimicrobial therapies could potentially lower the risk of toxicity and resistance development (Qvortrup et al., 2019).

Recently, we reported a 5-aryl-2-aminoimidazole (2-AI)-based antibiofilm coating specifically targeting biofilm formation (Peeters et al., 2019). The in-house discovered compound 1-(8-aminooctyl)-5-(4-bromophenyl)-*N*-cyclopentyl-1*H*-imidazol-2-amine (LC0024-NH_2_) was successfully linked to a titanium surface resulting in a reduction in biofilm formation without affecting the planktonic phase. The mechanism of action has been partially elucidated in a study on *Salmonella* (Robijns et al., 2014) where 2-AIs strongly reduced the expression of genes (i.e., *csgD*, *csgB*, and *adrA*) involved in the production of the EPS. Inhibition of public goods such as EPS, without affecting cell viability, is less likely to impose a strong selective pressure for resistance (Allen et al., 2014). Consistently, our *in vitro* evolution and competition experiments showed that strains resistant to 2-AIs are counter selected, proving this strategy to be resilient to resistance development (Dieltjens et al., 2020). Since the ability to form mature biofilms is hindered in the presence of 2-AIs, perioperative contamination is less likely to result in biofilm formation on the surface of the coated device. Moreover, this strategy has the potential to prevent the life-long risk of hematogenous ODRI (Rakow et al., 2019), as the active compound is covalently bonded to the implant surface and is not released, like is the case for many other implant coatings.

In the present study, we aimed to optimize the 2-AI coating protocol to achieve a higher surface loading, a more efficient synthesis process and a higher coating stability, both for titanium disks and commercially available titanium fracture fixation devices. In addition, we evaluated the compatibility of the coating with the most common sterilization procedures and performed an *in vivo* safety study in a rabbit fracture model to determine the impact of the coating on fracture healing.

## Materials and Methods

### Bacterial Strain and Chemicals

The bacterial strain *S. aureus* JAR060131, a clinical isolate originally cultured from a human patient with ODRI, was used in this study (culture collection of Switzerland number CCOS 890). Overnight cultures of *S. aureus* JAR060131 were grown in Lysogeny broth (LB), in test tubes at 37°C in shaking conditions. *In vitro* biofilm assays were performed using a 1/20 dilution of Tryptic Soy Broth (TSB 1/20). Phosphate-buffered saline (PBS) was prepared by combining 8.8 gL^–1^ NaCl, 1.24 gL^–1^ K_2_HPO_4_, and 0.39 gL^–1^ KH_2_PO_4_ (pH 7.4). LC0024-NH_2_ was prepared as previously reported (Peeters et al., 2019). All other chemicals were purchased from commercial sources and used without further purification.

### 2-AI Covalent Binding on Titanium Disks, Locking Compression Plates, and Screws

#### Coating Procedure

Round titanium disks (Ti6Al4V alloy, grade 5; height: 4 mm; diameter: 12 mm, total surface area: 3.77 cm^2^) were purchased from Salomon’s Metalen bv (Groningen, Netherlands) and used for coating optimization and *in vitro* activity testing. Prior to functionalization, the disks were roughened by bead blasting with high purity Al_2_O_3_ particles (Elysee Dental Belgium NV). The surface was cleaned and activated by chemical etching using a 20 wt% HNO_3_ and 4 wt% HF aqueous solution, followed by thorough washing with demineralized water and acetone. These disks without further functionalization are called control-Ti. To covalently bind the active compound, the surface was functionalized with amine groups by treatment with Fmoc-protected 3-aminopropyltriethoxy silane (Fmoc-APTES), followed by deprotection with tetrahydrofuran (THF)/piperidine (90:10) and thorough washing with THF. The silanization protocol was repeated three times. The hydrolysis solution was kept for the quantification of -NH_2_ groups *via* UV-Vis spectroscopy. After drying, the aminated disks were placed in a 15 ml falcon tube containing a n-hexane/hexamethylene diisocyanate (HMDI; 85:15) solution (1 mL/disk), agitated for 3 h at room temperature with the aid of a roller mixer and then rinsed with *n*-hexane. Next, the disks were transferred to a solution of LC0024-NH_2_ in DMSO, agitated for 16 h and rinsed with demineralized water and afterward with acetone. The rinsed samples were then dried at room temperature for 1 h. These disks are further referred to as LC0024-Ti. The titanium locking compression plates (LCPs) and screws, used in the *in vivo* study, were obtained from Depuy Synthes; Johnson & Johnson Co. Inc., NJ, United States. LCPs and screws were also etched and coated with LC0024-NH_2_, as described above. Control LCPs and screws did not receive any treatment.

#### Quantification of -NH_2_ Groups

The hydrolysis solutions from the Fmoc-deprotection procedure were used for quantification of the –NH_2_ groups by means of UV-Vis spectroscopy (Carey 5000, Varian, United States). To this end, the solution was diluted ten times with THF/piperidine (90:10) and the absorbance at 300 nm was measured against a pure THF/piperidine (90:10) solution.

#### LC0024-NH_2_ Loading Quantification

Quantification of the LC0024-NH_2_ loading was performed, as previously reported (Peeters et al., 2019), by hydrolysis and detachment from the linker followed by analysis of the hydrolysis solution *via* fluorescence spectroscopy. Briefly, the coated supports were fully immersed in the appropriate amount of hydrolysis solution composed of deionized water, isopropanol, and triethylamine in 1:0.5:1 volume ratio. The mixture was heated at 60°C for 1 h. Fluorescence absorbance of LC0024-NH_2_ of the hydrolysis solution was measured with a fluorescence spectrophotometer (FLS 920, Edinburgh Instruments, Photonics division) at excitation and emission wavelengths of 380 and 462 nm, respectively. The LC0024-NH_2_ loading was calculated according to a calibration curve obtained from serial dilutions of known standards in the hydrolysis solvent.

### Sterilization Procedures

The coated and non-coated LCPs and screws were double packed in sterilization foil (Steriking, Wipac medical, Bomlitz, Germany) and steam sterilized in a steam autoclave (Vapofix 3-3-6 VS1, Belimed, Zug, Switzerland). The sterilization cycle used saturated steam generated from de-ionized water and maintained at 134°C for 6 min at 3,100 mbar. The same protocol was applied to the titanium disks. Ethylene oxide sterilization of titanium disks was performed by 2 h exposure at 55°C and negative pressure (at least 80 mbar below atmospheric pressure).

### *In vitro* Biofilm Inhibition Assay and Antibiotic Co-Administration

To compare the *in vitro* antibiofilm activity of the LC0024-Ti disks with the control-Ti, the LC0024-Ti disks were inoculated with *S. aureus* for 24 h and the number of colony forming units (CFUs) on the surface of the disks (biofilm) and in the supernatant (planktonic) were determined. The disks were sterilized by immersion in ethanol solution for 10 min and dried under a laminar flow. Afterward, the disks were wrapped with Teflon tape and fitted snuggly into silicon rubber rings placed in the wells of a 12-well plate to prevent biofilm formation on the sides and bottoms. A first preconditioning step was performed by pouring 400 μL of bovine serum albumin (BSA, Sigma-Aldrich, Europe) in the wells, sealing the plate with a sterile semi-permeable membrane and wrapping it with parafilm to prevent evaporation, and incubating it overnight at 37°C in a closed plastic bag. Thereafter, the BSA solutions were removed, and the exposed surfaces washed with PBS. Overnight cultures of *S. aureus* were diluted in TSB 1/20 medium and 400 μL of a 1 × 10^4^ cells/mL suspension was poured over each disk and the plate was covered with a semipermeable membrane. After static incubation for 24 h at 37°C, the supernatants were removed, diluted and plated on LB plates. The disks were carefully washed with sterile PBS to remove loose cells and transferred to falcon tubes filled with 2 mL PBS. The tubes with the disks were vigorously vortexed for 1 min, sonicated for 10 min at 45,000 Hz in a water bath sonicator (VWR USC 300-T) and vortexed again for 1 min. The resulting bacterial suspensions were passed through a 25G needle to disrupt any remaining aggregates, diluted, and plated on LB plates. After 24 h of incubation at 37°C, the numbers of CFU/ml were determined by plate counting. Finally, CFU/cm^2^ values were calculated accordingly by multiplying with the dispersion volume (2 ml) and dividing by the area of the exposed surface (1,13 cm^2^). The *in vitro* activity of steam sterilized disks was assessed using the same protocol.

Additional biofilm experiments were performed as mentioned above, except for the addition of cefuroxime to evaluate the impact of the coating in the presence of a conventionally used antibiotic. In these experiments, after a 24-h incubation period, the supernatant was replaced with a 0,004 μg/ml cefuroxime solution in TSB 1/20 for the treated samples and regular TSB 1/20 for the control disks. A second incubation period of 24 h followed and afterward the disks were handled as described above.

### Visualization of Biofilm

Biofilms were grown onto control-Ti and LC0024-Ti disks as described above. The samples were prepared for scanning electron microscopy (SEM) by fixation with glutaraldehyde, as previously described (De Brucker et al., 2015). After removal of loosely attached cells by gentle immersion of the disks in PBS, the samples were fixed with 2.5% glutaraldehyde (2.5% glutaraldehyde in cacodylate buffer [0.1 M, pH 7.4]) for 30 min and rinsed 3 times with PBS. Dehydration was performed through a series of ethanol washes (30, 50, 70, and 90% ethanol for 20 min each) followed by soaking in 100% ethanol for 20 min – each three times – and drying. Finally, the surface of the samples was sputter-coated with Pt (Q150/S, Quorum Technologies) and analyzed by SEM, operated at standard high-vacuum settings, and using a 4.8-mm working distance and 5-keV accelerating voltage using back-scattered electron (BSE) imaging.

### Evaluation of Fracture Healing in a Rabbit Model

The impact of the LC0024 coating on fracture healing was evaluated *in vivo* in a rabbit model. The study was approved by the ethical committee of the canton of Grisons in Switzerland (approval number TVB_ 07_19). All procedures were performed in an Association for Assessment and Accreditation of Laboratory Animal Care International (AAALAC)-approved facility and according to Swiss animal protection law and regulations.

The model involved the creation of a mid-diaphyseal osteotomy in a rabbit humerus with plate fixation as previously described (Arens et al., 2015). The osteotomy was performed using a 0.44 mm Gigly saw (RISystem, Switzerland) and fixed with a 7-hole (coated or uncoated) titanium LCP and (coated or uncoated) six locking screws (central plate hole was left empty).

#### Animals

Nine skeletally mature female New Zealand white rabbits (Charles River, Sulzfeld, Germany) were used in this study. All animals underwent clinical examination and were found healthy prior to inclusion in this study. The animals were divided in two groups and implanted with coated (6 animals) or uncoated (3 animals) fracture fixation devices (LCPs). After 8 weeks, the animals were euthanized using intravenously administered pentobarbital (200 mg/kg; Esconarkon).

#### Clinical Observations

An animal caretaker checked the rabbits three times a day for the first post-operative week. Thereafter, all animals were monitored daily. Their general and eating behavior as well as weight-bearing on the operated leg was scored. The surgical incision, respiration, eyes, fur and feces were also monitored and logged. Blood samples were taken preoperatively, at day 3 and day 7 postoperatively, and were continued once a week thereafter. Blood and serum were used to measure white blood cell count (VET ABC, Scil animal care, Viernheim, Germany) and C-reactive protein (CRP; rabbit CRP ELISA Kit, ICL Inc. Portland, OR, United States), respectively. The weight of each animal was determined at the same timepoints. Radiographs of the operated limbs were taken in anteroposterior and lateral views postoperatively and every 2 weeks until euthanasia.

#### Histopathology

A post-mortem high-resolution contact radiograph was taken of the humerus of each animal in two directions (caudocranial and lateromedial). All humeri were fixed for a minimum of 2 weeks in 70% methanol. After fixation, samples were dehydrated through an ascending series of ethanol (70%, 96%, absolute ethanol) with two changes for each step, every 4–7 days. Samples were transferred to xylene and finally to methylmethacrylate (MMA) for embedding. The polymerized samples were trimmed on a butcher saw and glued to beracryl holders and sectioned using a Leica 1600 saw microtome. A high-resolution contact radiograph was taken of the sectioned bones. Sections were then stained with Giemsa Eosin. Imaging of the Giemsa Eosin stained sections was performed using brightfield illumination. A veterinary histopathologist performed a semi-quantitative histopathological analysis using a six-point grading system (grade 0: change absent, grade 1: minimal change, grade 2: slight change; grade 3: moderate change; grade 4: marked change; and grade 5: massive change). Median grades were calculated per group (coated vs. uncoated). Due to the different number of animals per group, the severity and incidence of changes was calculated per animal.

### Statistical Analysis

All *in vitro* experiments were carried out in technical triplicates and were independently repeated at least three times. Statistical significance of the *in vitro* data was determined by applying a two-sided ratio paired *t*-test using GraphPad Prism version 9 (GraphPad Software, United States).

## Results

### Improved Coating Procedure and Loading Analysis

**FIGURE 1:**
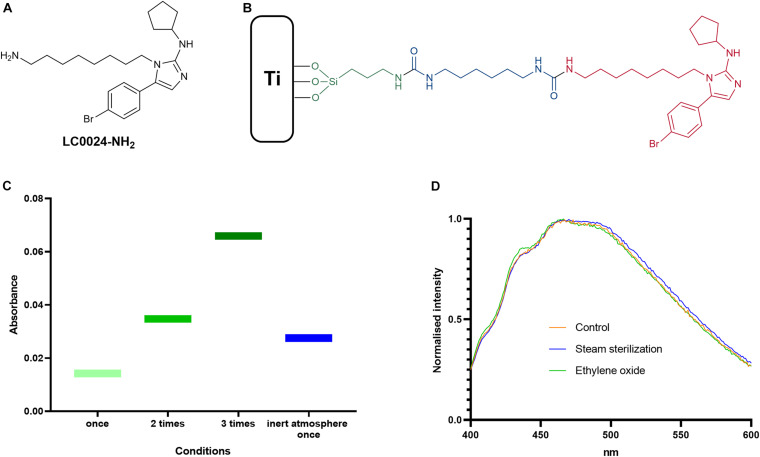
Optimized coating and chemical analyses. **(A)** Schematic structure of LC0024-NH_2_; **(B)** schematic representation of the coating sub-units; **(C)** results from the silanization step optimization; and **(D)** fluorescence emission profiles of the sterilized samples compared to the control disks.

An optimized protocol for the attachment of the active compound LC0024-NH_2_ (Figure 1A) to titanium surfaces was developed. A schematic representation of the coating is presented in Figure 1B. The first step of the coating procedure consisted in functionalizing the titanium surface with Fmoc-APTES. When the reaction was carried out in an inert atmosphere the loading showed a slight increase compared to standard conditions. Nonetheless, higher surface loading could be obtained by multiple repetitions of the coating procedure. The graph in Figure 1C shows the increased loading following the increased number of repetitions. After cleavage of the Fmoc groups, LC0024-NH_2_ was covalently bonded to the aminated surface using HMDI as a linker. This shortened the synthetic protocol with one step as compared to our previous report (Peeters et al., 2019). The amount of LC0024-NH_2_ was measured by fluorescence spectroscopy as described in the Materials and Methods and resulted in a final loading of 50 nmol/cm^2^. The presence of LC0024-NH_2_ was also confirmed by scanning electron microscopy with associated energy-dispersive X-ray spectroscopy (SEM-EDX) (Supplementary Table 2). Stability of the coating was evaluated against two common sterilization procedures, steam sterilization and exposure to ethylene oxide. Therefore, the LC0024-Ti disks were sterilized using both techniques. After sterilization, the active compound was detached from the titanium surface by hydrolysis and the resultant solutions were analyzed *via* fluorescence spectroscopy. The fluorescence emission profiles of the two sterilization procedures did not show a significant difference compared to the profile of the control disks (Figure 1D).

### *In vitro* Antibiofilm Activity Evaluation

**FIGURE 2:**
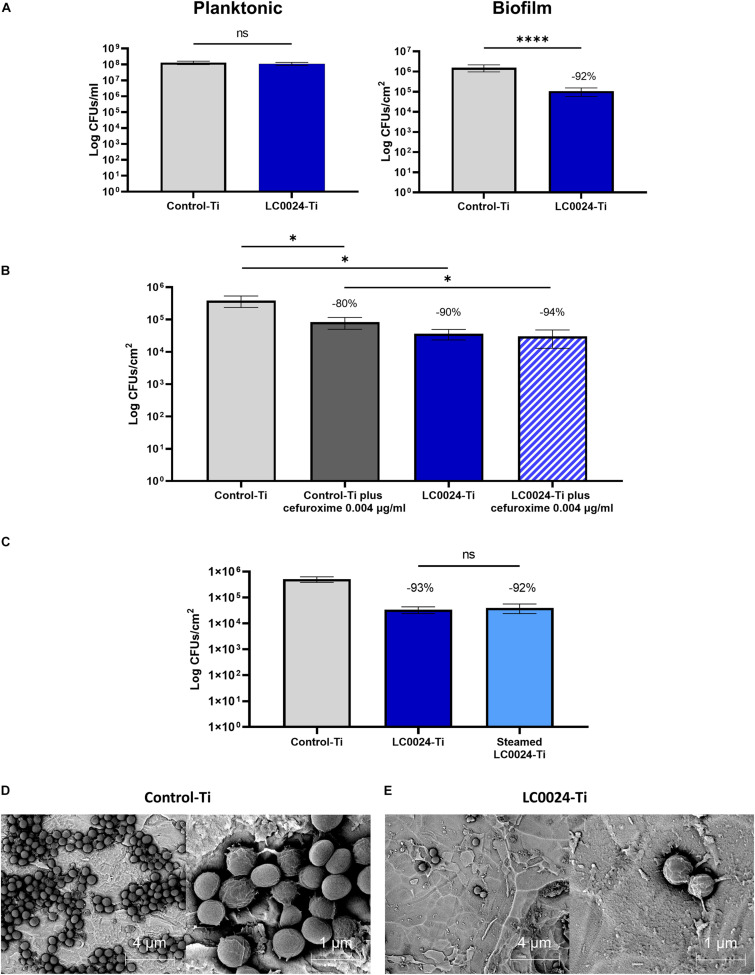
Overview of *in vitro* activity evaluation. **(A)** *In vitro* characterization of planktonic growth and biofilm formation of *S. aureus* JAR060131 on LC0024-Ti disks. Data represent the mean of at least three independent experiments, the error bars show the standard error of the mean (SE). Statistical significance was determined by applying a two-sided ratio paired *t*-test using GraphPad Prism (ns, not significant; ****p < 0.0001), the average relative reduction compared to the untreated Control-Ti is reported as percentage; **(B)** *In vitro* evaluation of the effect of low concentration of cefuroxime (0.004 μg/ml) on biofilm cells present on the surface of the LC0024-Ti disks as compared to the control-Ti disks. Data represent the mean of three independent experiments, the error bars show the standard error of the mean (SE). Statistical significance was determined by applying two-sided ratio paired *t*-test using GraphPad Prism (*p < 0.05), the average relative reduction compared to the untreated Control-Ti is reported as percentage; **(C)** *In vitro* evaluation of the effect of steam sterilization on the LC0024-Ti disks as compared to the control-Ti disks and non-sterilized LC0024-Ti disks. Averages of biofilm cells from three technical replicates are reported. Data represent the mean of three independent experiments, the error bars show the standard error of the mean (SE). Statistical significance was determined by applying a two-sided ratio paired *t*-test using GraphPad Prism (ns, not significant), the average relative reduction compared to the untreated Control-Ti is reported as percentage; **(D)** Representative SEM-BSE images of a fixed biofilm grown on control-Ti disks; and **(E)** Representative SEM-BSE images of a fixed biofilm grown on LC0024-Ti disks.

After optimizing the coating procedure, we continued with *in vitro* evaluation of the antibiofilm activity. In order to provide relevant data for future *in vivo* studies, a clinical isolate of *S. aureus* (JAR06.01.31), was chosen. As shown in Figure 2A, biofilm formation after 24 h was significantly affected with an average decrease of biofilm cells with 1 log (92% reduction; remaining cells: 1,06 × 10^5^ CFU/cm^2^) for LC0024-Ti compared to the control-Ti, whilst planktonic cells were not affected. Similarly, SEM pictures, of fixed biofilms showed a strong difference in biofilm growth (Figures 2D,E). The surface of LC0024-Ti disks was populated mainly by isolated cells whereas aggregates of biofilm cells were visible on the control-Ti disks (Figures 2D,E). This finding is in accordance with the described mechanism of action of 2-AI, i.e., inhibiting matrix production (Robijns et al., 2014; Peeters et al., 2019).

A second set of experiments was carried out to evaluate the combined activity of the coating and a classic antibiotic. Biofilms were set to grow for 24 h on coated and uncoated titanium disks, after which the biofilms were submerged in a cefuroxime solution at 0.004 μg/ml in TSB 1/20 for an additional 24 h. For the untreated control group, the supernatant was replaced with fresh TSB 1/20. The treatment with cefuroxime alone caused a 80% reduction in biofilm cells (remaining cells: 8.28 × 10^4^ CFU/cm^2^; Figure 2B) while the coating alone caused a 90% reduction (remaining cells: 3,64 × 10^4^ CFU/cm^2^) demonstrating resilience to biofilm formation also after 48 h. The combined effect of the two treatments resulted in a significant (*p* = 0.0498) further reduction of 14% (-5.28 × 10^4^ CFU/cm^2^) compared to cefuroxime alone and the remaining biofilm cells accounted for only ∼6% (94% reduction; remaining cells: 3.02 × 10^4^ CFU/cm^2^) compared to the control. The activity of the antibiotic against cells on the Control-Ti surface [79.45% ± 6.17 (SE) reduction upon cefuroxime treatment] and the LC0024-Ti surface [24.81% ± 36.91 (SE) reduction upon cefuroxime treatment] did not significantly differ (*p* = 0.2182). Before attempting the *in vivo* experiments, activity retention after sterilization was tested *in vitro* using steam sterilized disks. The results showed that activity was retained (93 and 92% reduction for LC0024-Ti and steamed sterilized LC0024-Ti disks, respectively; Figure 2C).

### Histopathological and Radiographical Evaluation

**FIGURE 3:**
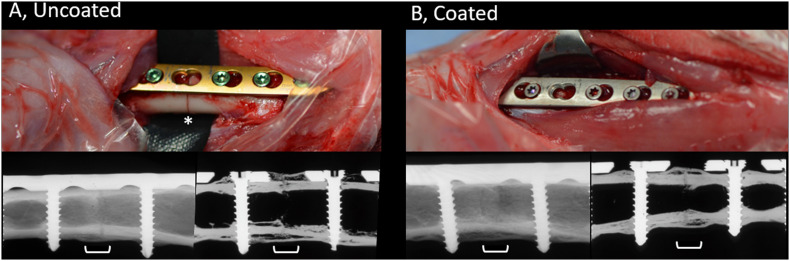
Appearance of uncoated **(A)** and 2-AI-based coated **(B)** plates during placement and radiographic appearance of healing at time of study completion. Upper row, intra-operative pictures of the surgical site prior to wound closure showing LCPs being fixed to the bone. Skeletally mature rabbits received a 7-hole LCP and six screws. Note the position of the osteotomy beneath the empty screw hole on left hand side image (asterisk). The uncoated plates and screws are as purchased with coloring due to anodization. The color of the coated plates and screws match, due to the coating process that was applied. Lower row, post-mortem contact radiographs of the bone (left) or after sectioning (right), centered on the osteotomy (white bracket). Note the complete bridging of the osteotomy gap and the formation of periosteal callus at the osteotomy site. The osteotomy is still visible indicative of ongoing healing. Images were randomly selected as representative of the entire group.

The contact radiographs of rabbits in the coated and uncoated groups 8 weeks after osteotomy are shown in Figure 3. In the uncoated group (Figure 3A), where rabbits received commercially available medical grade titanium LCPs, healing progressed well and there was bridging of the osteotomy and a physiological presence of periosteal callus at the osteotomy site. Similarly, rabbits in the coated group (Figure 3B) also displayed healing of the osteotomy and a similar periosteal callus appearance as the uncoated group. In both the coated and uncoated groups, healing was not yet totally completed, as revealed by sections through the bone (lower right-hand image in Figures 3A,B), and no differences were observed between the groups based on visual inspection of contact radiographs.

**FIGURE 4:**
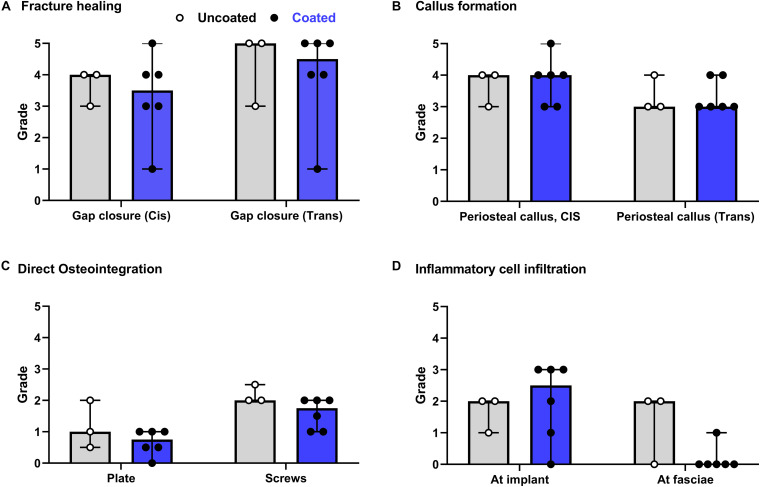
Overview of histological evaluation of rabbits receiving coated and uncoated screws. **(A)** Fracture healing; **(B)** callus formation; **(C)** direct osteointegration; **(D)** inflammatory cell infiltration. Rabbits were euthanized 8 weeks after osteotomy and fixation with uncoated (gray bars [median/group], white dots [individual values]) or coated (blue bars, black dots) LCPs and screws. The entire operated bone was fixed, embedded in polymethylmethacrylate (PMMA), sectioned and stained with Giemsa Eosin. Semi-quantitative histological scores (0–5) were given by a blinded veterinary pathologist for a group of *n* = 6 rabbits receiving coated implants and *n* = 3 rabbits receiving uncoated implants. Features were scored for the entire section. Error bars represent the range. No statistical comparisons made.

The semi-quantitative histological evaluation of coated/non-coated plates and screws and the local tissues is shown in Figure 4. The data points from each evaluation showed a rather homogenous response among the different animals. The few scores that notably differ are due to inherent variability of the model as they are not attributable to the discordant response of one single animal. This variability can be associated with mechanical effects caused by minor differences in plate and screw placement, as well as intrinsic limitations in the sampling method whereby a three-dimensional object is scored on a single longitudinal section. In general, the score for fracture healing (Figure 4A) shows equivalent healing in both groups at both the *cis* and *trans* cortex, consistent with the radiographic appearance shown in Figure 3. Similarly, periosteal callus (Figure 4B) at both the *cis* and *trans* side were similar between groups, and at a moderate to high grade consistent with the radiographic signs. A moderate increase of cortical porosity near the osteotomy site and a minimal to moderate cortical thinning (attributed to stress-shielding) was found in both groups.

**FIGURE 5:**
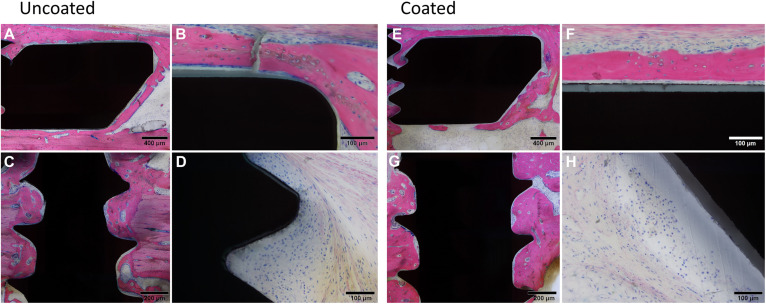
Histological images of rabbits receiving Left, uncoated, or Right, coated LCPs and screws. At euthanasia, 8 weeks after surgery, rabbits were submitted for histopathological processing and evaluation. Giemsa Eosin-stained sections of the plate **(A,E)** and screws **(C,G)** are shown that are indicative of typical findings. **(B,F)** show higher magnification images of **(A,E)**, respectively. Note the greater bone-implant contact for the screws relative to the plates and a lack of significant differences between the groups. Low to moderate grade infiltration with inflammatory cells was also observed (uncoated, **D** and coated **H**) again without marked differences between the groups. Images were selected as representative of features observed.

Direct osteointegration (segments with direct bone-implant contact) was also comparable between both groups, with a relatively low score in both groups for both the plates and screws (Figure 4C). Microscopic images of the Giemsa Eosin-stained sections show the osteointegration of plates and screws in the uncoated group [Figure 5A (magnified in B) and C, respectively] and in the coated group [Figure 5E (magnified in F) and G]. There was greater direct bone-implant contact around the screws compared to the plates, without any marked differences between the coated and uncoated groups.

The severity of the inflammatory cell infiltration was also scored (Figure 4D) and microscopic images are shown in Figures 5D,H for the uncoated and coated groups, respectively). Any observed inflammation was either serous (formation of fluid- and cell-filled space) and/or mononuclear (lymphoplasmacellular cell infiltration). Generally, it was relatively low and broadly equivalent between both groups. No inflammatory cell infiltration was recorded in the osteotomy gap or bone marrow (not shown).

## Discussion

The eradication of ODRI is challenging due to the presence of a biofilm that forms a physical and chemical barrier, thereby protecting bacteria from antibiotics and host defenses. Moreover, reduced metabolic activity of bacteria within the biofilm further reduces the activity of certain antibiotics. Biofilms also offer protection against phagocytes by offering mechanical protection and preventing the engulfment process as described for *S. aureus* (Thurlow et al., 2011). In addition, treatment of ODRI becomes challenging when compromised vascularization at the surgical site hampers the penetration of antibiotics and vital components of the immune system. Therefore, biofilm formation should be considered the key target in tackling ODRI. As an example, surface modification by covalent attachment of an antibiofilm-specific compound has shown to prevent ODRI by inhibiting bacterial colonization while on the other hand allowing tissue integration (Seebach and Kubatzky, 2019).

This study presents our latest progress regarding the development of an antibiofilm coating for titanium implants *via* covalent binding of 2-AI LC0024-NH_2_. Recently, we reported our first results regarding the application of 2-AI in the prevention of *S. aureus* biofilm formation on titanium surfaces (Peeters et al., 2019). *In vitro* experiments and *in vivo* data in a biomaterial-associated murine infection model proved the retention of activity of the LC0024-NH_2_ when covalently bonded onto titanium. In the present work we have optimized the coating procedure by shortening the synthetic route by one step, and achieved a 5-fold increase in surface density as compared to our earlier results. The previously adopted procedure for covalent binding LC0024-NH_2_ onto the titanium surface consisted of functionalization with carboxylic moieties followed by amide coupling with LC0024-NH_2_ *via* a four step synthesis (Peeters et al., 2019). Specifically, amination of the titanium oxide layer was performed *via* a single step of silanization using APTES. The aminated surface was then reacted with HMDI, followed by reaction with a 6-aminohexanoic acid solution for 16 h. HMDI hereby acted as a linker between aminopropyl silane and 6-aminohexanoic acid. Finally, LC0024-NH_2_ was bonded *via* amide synthesis to the carboxylic moieties hanging from the surface. This resulted in a LC0024-NH_2_ loading of 10.1 nmol/cm^2^. In the present study, we attempted to improve several steps in this coating procedure and increase the loading. We started with the optimization of the silanization step. Literature studies suggested that an inert atmosphere could have a positive effect on the attachment of silanes onto surfaces (Zhu et al., 2012). Degassing and purging nitrogen gas into the Fmoc-APTES solution indeed caused a slight increase in loading (Figure 1C). Nonetheless, simple repetition of the standard procedure without use of an inert atmosphere resulted in an even higher surface density. To simplify later upscaling of the coating procedure, the inert atmosphere option was not further adopted. Furthermore, since LC0024-NH_2_ could potentially react directly with HMDI without the need for adding the aminoacidic linker, we speculated that this lengthy step could be avoided. This was indeed confirmed by the observed increase in overall yield. Moreover, the absence of the hydrolysable amide bond associated with the aminoacidic linker can have the additional benefit of making the coating more resistant to deactivation processes (i.e. chemical or enzymatic hydrolysis) and thus retaining activity for a longer period. Nonetheless, reduction in the chain length, and possibly flexibility, could have an effect on the biological activity. Therefore, *in vitro* experiments were carried out to ensure retention of activity.

*In vitro* antibiofilm activity of the newly optimized LC0024-Ti disks was assessed against the clinical *S. aureus* isolate JAR06.01.31. The number of biofilm cells onto LC0024-Ti disks surface was reduced on average with more than 90% compared to the uncoated control-Ti disks. In our previous report, LC0024-NH_2_ coating led to a 47% reduction in biofilm cells (Peeters et al., 2019). These results also confirmed that the reduction in length of the chain was not detrimental to activity. As the viability of planktonic cells was not significantly affected, this enhanced activity solely targeted biofilm formation. To gain further insights in our strategy, we compared the effect of antibiotic treatment on biofilms grown onto LC0024-Ti and control-Ti disks. Since 2-AIs target EPS production, the integrity of the biofilm matrix is expected to be compromised (Mongkolrob et al., 2015). Consequently, antibiotic treatment might show a stronger effect in the presence of the coating. This would have a beneficial impact on the resolution of ODRIs allowing for shorter antibiotic therapies at lower concentrations. Cefuroxime was the antibiotic of choice in this study as it is commonly employed in daily clinical practice and well tolerated by rabbits. We selected a cefuroxime concentration of 0.004 μg/ml that is much lower than the MBC and MBEC (Supplementary Table 3), because at such suboptimal antibiotic concentration the tolerance effects of the biofilm, and thus potential effects of the antibiofilm coating on antibiotic efficacy, are expected to be most pronounced. We observed a similar activity of the antibiotic against bacterial cells on the antibiofilm coated and uncoated surface. The combined effect of cefuroxime with the coating (94% reduction compared control-Ti) was therefore higher than that of cefuroxime alone (80% reduction compared to control-Ti). The anticipated higher efficacy of the antibiotic against cells on the anti-biofilm surface, which we previously reported for *Salmonella* (Dieltjens et al., 2020), was, however, not observed. This might be related to the difference in bacterial target species, but also to the longer antibiotic treatment period in the current experiment [24 h vs 1 h in our previous report (Dieltjens et al., 2020)] which might have introduced additional effects such as partial antibiotic degradation, re-attachment of dispersed bacterial cells and/or antibiotic resistance. The current study focused on effects of the coating on early colonization and early stage biofilm formation (up to 48 h). In future studies we plan to implement flow-cell devices for biofilm formation and monitoring. Continuous flow of fresh medium ensures the constant supply of nutrients for prolonged periods of time. This will allow us to study the effect of the coating on biofilm maturation. Moreover, we aim to extend our focus on other clinically relevant strains and species (e.g., *S. epidermidis).*

Before proceeding with the preparation of the LCPs for the *in vivo* evaluation, stability of the coating to conventional sterilization procedures was assessed. Heat treatment as well as chemical sterilization can affect coating activity by hydrolysis of the active compound or the linker (Duncan et al., 1998; Preem et al., 2019). Other undesired reactions such as oxidation or radical formation can also compromise the coating activity. The coating was therefore challenged with steam sterilization and exposure to ethylene oxide, two commonly used procedures for implant sterilization. Analysis of the hydrolysis solutions from sterilized samples did not show significant variations compared to the untreated LC0024-Ti disks. Moreover, antibiofilm activity of steam sterilized LC0024-Ti disks was confirmed *in vitro*. These results are of crucial importance prior to preclinical validation in living animals. Finally, the coating procedure was applied to LCPs and screws and the presence of the active compound on the surface was confirmed by fluorescence spectrophotometry.

Implant biocompatibility is of crucial importance for the success of orthopedic procedures and patient outcome. Although it is true that fracture fixation devices may be removed after fracture healing, which makes direct osteointegration less important, biomechanical stability of the implant plays a crucial role in fracture healing. Moreover, stability is an important aspect with respect to prevention and treatment of infection, due to a vicious cycle between instability, ongoing soft tissue trauma and osteolysis (Foster et al., 2021). Stability of the fractured bone, that is provided by fracture fixation devices (e.g., plates and screws), requires a biocompatible surface that does not lead to inflammation or loss of local bone (i.e., osteolysis) at the bone-implant interface. The interaction of host and implant is significantly impacted by the surface of the implant and is first of all influenced by an initial conditioning layer of host proteins, followed later by fibroblast adhesion and later bone forming cells (osteoprogenitor cells and osteoblasts; Nuss and Rechenberg, 2008). Interference with any of these phases due to physicochemical properties of the implant surface can undermine the success of the surgery, and, therefore, needs careful evaluation. In our previous study, evaluation of the *in vitro* osteointegration potential [use of human bone marrow-derived stromal cells (MSC) and human microvascular endothelial cells (HMVEC)] and *in vivo* osteointegration in a rat model showed a positive outcome (Peeters et al., 2019). To further validate the applicability, the current study provides evidence for the biocompatibility of the coating in a rabbit fracture model. Histological evaluation showed that the grade of osteotomy gap closure and of periosteal callus formation were high and comparable for both groups. This confirms that the 2-AI coating does not interfere with fracture healing. Furthermore, both groups showed a low and similar amount of inflammatory cell infiltration.

In conclusion, this study reported on an optimized coating procedure to covalently bind the antibiofilm compound LC0024-NH_2_ on titanium surfaces. This resulted in higher loading and an enhanced activity *in vitro*. When applied to fracture fixation devices the coating did not negatively affect fracture healing in a rabbit fracture model. These positive results pave the way for future translational studies where we aim to confirm the effectiveness of the optimized coating against infection *in vivo.* Furthermore, they put our technology one step closer to clinical trials, confirming its potential in fighting ODRI without inducing local toxicity.

## Data Availability Statement

The raw data supporting the conclusions of this article will be made available by the authors, without undue reservation.

## Ethics Statement

The animal study was reviewed and approved by ethical committee of the canton of Grisons in Switzerland (approval number TVB_ 07_19).

## Author Contributions

GC performed the synthesis, chemical analysis, and *in vitro* activity tests. JO prepared and contributed to the surgeries and performed dissections. SZ and CC performed the surgeries. MK performed SEM analysis. DN performed the histopathological and radiographical evaluation. GC, JO, TM, HS, and W-JM wrote and revised the manuscript. EV, CC, SZ, and AB revised the manuscript. EV, HS, and W-JM contributed to project administration and funding acquisition. All authors contributed to the article and approved the submitted version.

## Conflict of Interest

The authors declare that the research was conducted in the absence of any commercial or financial relationships that could be construed as a potential conflict of interest.

## Supplementary Material

Supplementary Table 1 SEM-EDX analysis of control-Ti.

Supplementary Table 2 SEM-EDX analysis of LC0024-Ti.

Supplementary Table 3 Susceptibility of *S. aureus* JAR060131 to cefuroxime.
