# Supplementary material for: An Improved 2-Aminoimidazole Based Anti-Biofilm Coating for Orthopedic Implants: Activity, Stability, and in vivo Biocompatibility
Source: Front Microbiol. 2021 Apr 21;12:658521. doi: 10.3389/fmicb.2021.658521 (PMC8097006; doi:10.3389/fmicb.2021.658521)
Supplement: Supplementary Table 1 — SEM-EDX analysis of control-Ti. [file Table_1.docx]

**SUPPLEMENTARY MATERIAL**

**SEM/EDX analysis**

The control-Ti and LC0024-Ti surfaces were examined by scanning electron microscopy (SEM, Nova NanoSEM 450, FEI) with associated energy-dispersive X-ray spectroscopy (EDX, EDAX). All samples were sputtered (Q150/S, Quorum Technologies) with Pt prior to SEM analysis. The beam damage of the organic coatings was minimized using a low accelerating voltage of 5 keV. Immersion mode was applied to increase the resolution of the SEM images.

| 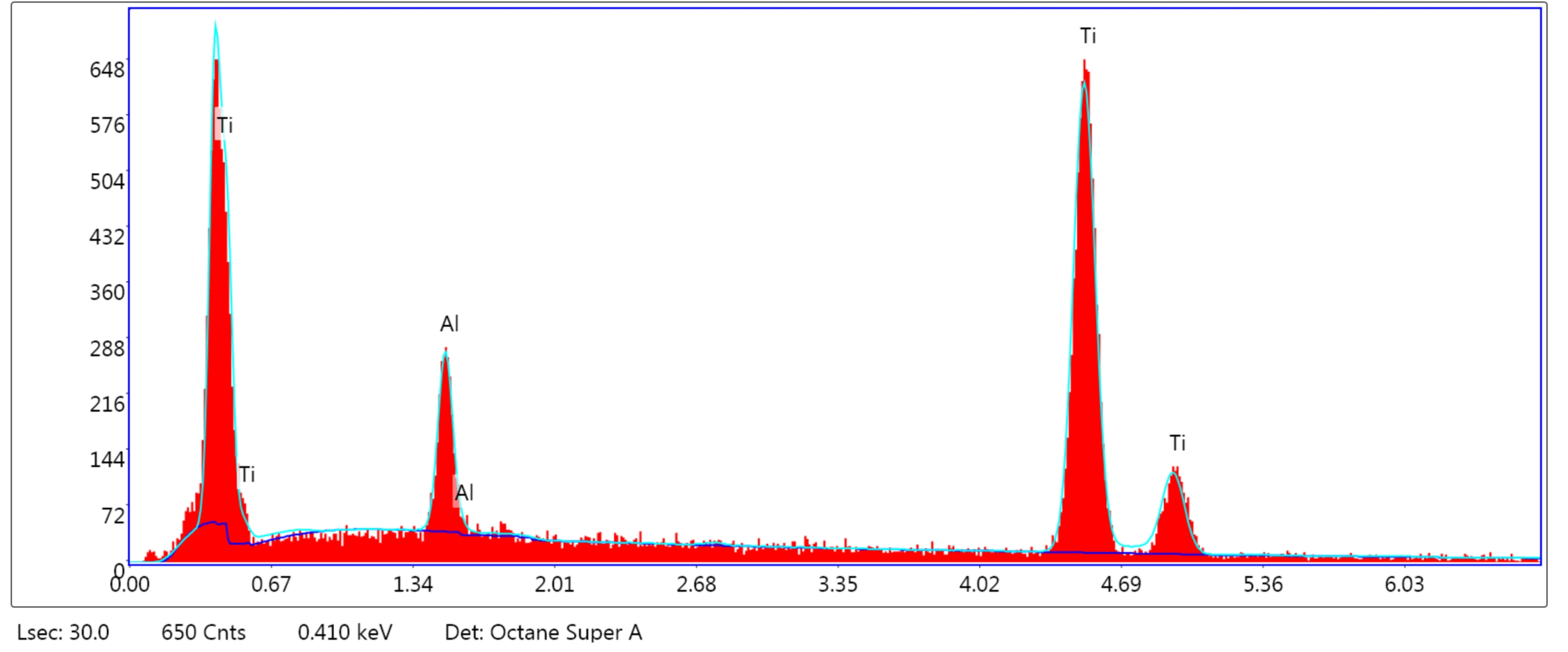 | | | | | | | | | | | | | | | | | | | | | | | | | | | | | | | | | | | | | | | |  |  |
| --- | --- | --- | --- | --- | --- | --- | --- | --- | --- | --- | --- | --- | --- | --- | --- | --- | --- | --- | --- | --- | --- | --- | --- | --- | --- | --- | --- | --- | --- | --- | --- | --- | --- | --- | --- | --- | --- | --- | --- | --- | --- |
|  |  |  |  |  |  |  |  |  |  |  |  |  |  |  |  |  |  |  |  |  |  |  |  |  |  |  |  |  |  |  |  |  |  |  |  |  |  |  |  |  |  |
|  |  |  |  |  |  |  |  |  |  |  |  |  |  |  |  |  |  |  |  |  |  |  |  |  |  |  |  |  |  |  |  |  |  |  |  |  |  |  |  |  |  |
|  |  |  |  |  |  |  |  |  |  |  |  |  |  |  |  |  |  |  |  |  |  |  |  |  |  |  |  |  |  |  |  |  |  |  |  |  |  |  |  |  |  |
| **eZAF Smart Quant Results** | | | | | | | | | | | | | | | | | | | | | | | | | | | | | | | | | | | | | | | | | |
|  |  |  |  |  |  |  |  |  |  |  |  |  |  |  |  |  |  |  |  |  |  |  |  |  |  |  |  |  |  |  |  |  |  |  |  |  |  |  |  |  |  |
|  |  | Element | | | | Weight % | | | | Atomic % | | Net Int. | | | | Error % | | | | Kratio | | | | Z | | | | A | | | | | | F | | | | |  |  |  |
|  |  | AlK | | | | 5.50 | | | | 9.37 | | 70.57 | | | | 8.03 | | | | 0.0489 | | | | 1.1510 | | | | 0.7704 | | | | | | 1.0028 | | | | |  |  |  |
|  |  | TiK | | | | 94.50 | | | | 90.63 | | 272.39 | | | | 5.04 | | | | 0.9352 | | | | 0.9905 | | | | 0.9989 | | | | | | 1.0003 | | | | |  |  |  |
|  |  |  |  |  |  |  |  |  |  |  |  |  |  |  |  |  |  |  |  |  |  |  |  |  |  |  |  |  |  |  |  |  |  |  |  |  |  |  |  |  |  |

**Supplementary Table 1:** **SEM-EDX analysis of control-Ti.**

|  |  |  |  |  |  |  |  |  |  |  |  |  |  |  |  |  |  |  |  |  |  |  |  |  |  |  |  |  |  |  |  |  |  |  |  |  |  |  |  |  |  |  |  |  |  |  |  |
| --- | --- | --- | --- | --- | --- | --- | --- | --- | --- | --- | --- | --- | --- | --- | --- | --- | --- | --- | --- | --- | --- | --- | --- | --- | --- | --- | --- | --- | --- | --- | --- | --- | --- | --- | --- | --- | --- | --- | --- | --- | --- | --- | --- | --- | --- | --- | --- |
| 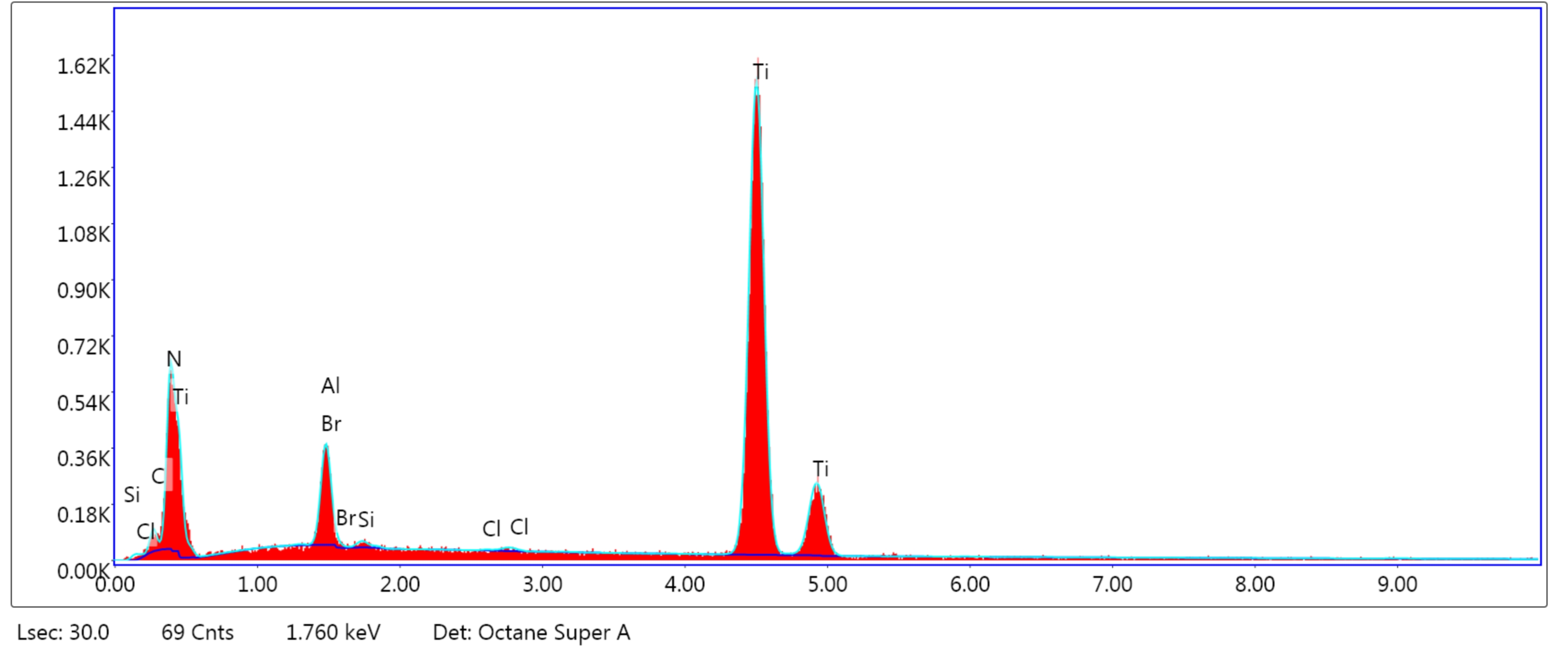 | | | | | | | | | | | | | | | | | | | | | | | | | | | | | | | | | | | | | | | | |  |  |  |  |  |  |  |
|  |  |  |  |  |  |  |  |  |  |  |  |  |  |  |  |  |  |  |  |  |  |  |  |  |  |  |  |  |  |  |  |  |  |  |  |  |  |  |  |  |  |  |  |  |  |  |  |
|  |  |  |  |  |  |  |  |  |  |  |  |  |  |  |  |  |  |  |  |  |  |  |  |  |  |  |  |  |  |  |  |  |  |  |  |  |  |  |  |  |  |  |  |  |  |  |  |
|  | |  |  |  |  |  |  |  |  |  |  |  |  |  |  |  |  |  |  |  |  |  |  |  |  |  |  |  |  |  |  |  |  |  |  |  |  |  |  |  |  |  |  |  |  |  |  |
|  | |  |  |  |  |  |  |  |  |  |  |  |  |  |  |  |  |  |  |  |  |  |  |  |  |  |  |  |  |  |  |  |  |  |  |  |  |  |  |  |  |  |  |  |  |  |  |
|  | |  | Element | | | | Weight % | | | | Atomic % | | Net Int. | | | | Error % | | | | Kratio | | | | Z | | | | A | | | | | | F | | | | |  |  |  |  |  |  |  |  |
|  | |  | C K | | | | 3.41 | | | | 11.10 | | 15.34 | | | | 17.09 | | | | 0.0154 | | | | 1.3070 | | | | 0.3463 | | | | | | 1.0000 | | | | |  |  |  |  |  |  |  |  |
|  | |  | N K | | | | 3.92 | | | | 10.94 | | 29.38 | | | | 14.44 | | | | 0.0222 | | | | 1.2767 | | | | 0.4437 | | | | | | 1.0000 | | | | |  |  |  |  |  |  |  |  |
|  | |  | BrL | | | | 2.18 | | | | 1.07 | | 19.38 | | | | 16.65 | | | | 0.0151 | | | | 0.8710 | | | | 0.7970 | | | | | | 1.0005 | | | | |  |  |  |  |  |  |  |  |
|  | |  | AlK | | | | 4.53 | | | | 6.56 | | 72.26 | | | | 9.36 | | | | 0.0320 | | | | 1.1105 | | | | 0.6348 | | | | | | 1.0028 | | | | |  |  |  |  |  |  |  |  |
|  | |  | SiK | | | | 0.30 | | | | 0.42 | | 5.27 | | | | 66.63 | | | | 0.0024 | | | | 1.1347 | | | | 0.7066 | | | | | | 1.0048 | | | | |  |  |  |  |  |  |  |  |
|  | |  | ClK | | | | 0.08 | | | | 0.09 | | 1.11 | | | | 80.24 | | | | 0.0008 | | | | 1.0565 | | | | 0.9110 | | | | | | 1.0208 | | | | |  |  |  |  |  |  |  |  |
|  | |  | TiK | | | | 85.57 | | | | 69.82 | | 652.03 | | | | 2.64 | | | | 0.8281 | | | | 0.9692 | | | | 0.9977 | | | | | | 1.0009 | | | | |  |  |  |  |  |  |  |  |

**Supplementary Table 2:** **SEM-EDX analysis of LC0024-Ti.**

**MIC, MBC and MBEC assays**

The minimum inhibitory concentration (MIC) and minimum bactericidal concentration (MBC) values of cefuroxime against *S. aureus* JAR060131 planktonic cells were determined using the broth microdilution method. A two-fold serial dilution of cefuroxime was prepared in a 96-well microtiter plate (100 μL per well; concentration range: 500 - 0,00095 µg/ml). Overnight cultures of *S. aureus* were diluted in TSB medium and 100 μL of a 1 × 10^6^ cells/mL bacterial suspension was added into wells. The plate was covered with a semipermeable membrane and incubated in a sealed bag at 37 ◦C for 20–24 h in shaking conditions (200 rpm). The MIC value was considered the lowest concentration that showed no visible growth. MBC was determined by inoculating 10 µL-aliquots from clear wells on TSB agar plates. The plates were incubated at 37 ◦C for 24 h. The MBC value was considered the lowest concentration producing a 99.9% CFU/ml reduction. A negative control, containing sterile broth and bacterial suspension (no antibiotic), and a sterility control containing only growth medium were included.

The minimum biofilm eradication concentration (MBEC) assay was performed using a 96-well microtiter plate growing the biofilm on the bottom of the wells. Overnight cultures of *S. aureus* were diluted in TSB medium and 200 μL of a 1 × 10^6^ cells/mL bacterial suspension was added into the wells. The plate was covered with a semipermeable membrane and incubated in a sealed bag at 37 ◦C for 24 h in static conditions. After incubation, the planktonic phase was removed, and the biofilm treated with serial two-fold serial dilution of cefuroxime in TSB (200 μL per well; concentration range: 50 - 0,000095 mg/ml). The plate was covered with a semipermeable membrane and incubated in a sealed bag at 37 ◦C for 24 h in static conditions. After incubation the planktonic phase was removed, and the wells were rinsed twice with sterile PBS (200 μL per well). Sterile PBS (200 μL per well) was added to the wells and the plate was covered with aluminum seal. The plate was placed in a water bath sonicator for 10 min at 45,000 Hz. Aliquots of 10 μL from each well were spread on TSB agar plates and incubated at 37 ◦C for 24-48 h. The MBEC value was considered the lowest concentration that showed no growth after incubation.

|  | MIC µg/ml | MBC µg/ml | MBEC µg/ml |
| --- | --- | --- | --- |
|  |  |  |  |
| *S. Aureus* (JAR060131) | 2 | 15,6-7,8 | >50000 |

**Supplementary Table 3:** **Susceptibility of *S. aureus* JAR060131 to cefuroxime.**
